# Teaching old mice new tricks: the utility of aged mouse models of C. difficile infection to study pathogenesis and rejuvenate immune response

**DOI:** 10.1080/19490976.2021.1966255

**Published:** 2021-08-25

**Authors:** Jae Hyun Shin, Sean W. Pawlowski, Cirle A. Warren

**Affiliations:** a Division of Infectious Diseases and International Health, University of Virginia, Charlottesville, VA, USA; b Colorado Infectious Disease Associates, Denver, CO, USA

**Keywords:** Animal model, aging, *Clostridioides difficile* infection, neutrophil response, microbiota

## Abstract

**Background:**

*Clostridioides difficile* is a serious problem for the aging population. Aged mouse model of *C. difficile* infection (CDI) has emerged as a valuable tool to evaluate the mechanism of aging in CDI.

**Methods:**

We reviewed five published studies utilizing aged mice (7–28 months) for CDI model for findings that may advance our understanding of how aging influences outcome from CDI.

**Results:**

Aged mouse models of CDI uniformly demonstrated more severe disease in the old compared to young mice. Diminished neutrophil recruitment to intestinal tissue in aged mice is the most consistent finding. Differences in innate and humoral immune responses were also observed. The effects of aging on the outcome of infection were reversed by pharmacologic or microbiota-targeted interventions.

**Conclusion:**

The aged mouse presents an important in vivo model to study CDI and elucidate the mechanisms underlying advanced age as an important risk factor for severe disease.

## Aging and CDI

*Clostridioides difficile* (formerly *Clostridium difficile*) is the most prevalent pathogen leading to healthcare-associated infections in the United States,^1^ accounts for an excess of a billion dollars to the healthcare system annually,^2^ and has been identified as one of the top five antibiotic resistance threats by the CDC.^3^ What is more striking is the toll on the aging population. *C. difficile* infection (CDI) is more likely to infect older patients,^4^ result in more recurrences,^5^ and more likely to end in death.^6^ Although people 65 and older make up half of the CDI patients, around 90% of deaths from CDI occur in this age group.^6^ Older patients also have poorer response to treatment for CDI.^7^ Even when controlling for confounding factors such as comorbidities and healthcare exposures, aging is an important factor to increase risk of worse outcomes.^8^ As the proportion of older people increases in the general population, it is even more compelling to pursue research evaluating the effect of age on CDI.

Animal models have long been used for *in vivo* research into pathogenesis of disease as well as for testing new treatment strategies. Animal models have been used to study the process of aging itself, utilizing a range of animals from *Caenorhabditis elegans, Drosophila melanogaster*, to mice and rats, up to primates.^9^ Rodent models have been used extensively due to the wealth of background knowledge, convenience of use, genetic manipulability, and expense.^9^ The rodent models of aging paved the way for aging research, such as the importance of caloric restriction on extending lifespan^10^ as well as for findings of certain genetic mutations being associated with longevity.^11^ Most studies have focused on conditions that accelerate aging or on measures that delay aging and increase longevity.^9^ In a limited capacity, aged mouse models have been used for infectious disease research as well. Especially in influenza infection, the aged mice have been used to test specific aspects of the pathogenesis,^12^ test new therapeutics,^13^ and test vaccines.^14,15^ However, the aged animal model was not utilized for research on CDI until recently. Over the past few years, studies utilizing aged animal models of CDI have been developed and published.^16–20^ The main challenge in development of an animal model of CDI is the microbiome. As seen in human disease, the normal intestinal microbiota must be disrupted before colonization and infection by *C. difficile* can occur.^21^ The development of varying models reflect the different methods utilized to cause susceptibility to *C. difficile* infection. One method would be to utilize gnotobiotic mice that do not possess a normal microbiome,^16^ while another method would be to utilize antibiotics to disrupt the normal intestinal microbiome.^17–20^ Different antibiotic regimens (combination antibiotic cocktail,^17–19^ and cefoperazone^20^) were utilized in different models, while the age of the mice also differed from model to model. In this paper, we reviewed the features of each animal model and the key findings from the studies. The characteristics of each animal model and the discoveries on the changes in response with aging are summarized in Table 1.

**Table 1.** Summary of effect of aging seen in aged mouse model studies

Study	Mechanism of susceptibility to infection	Age and sex of mice	Innate immune response			Humoral response	Microbiome
			Cellular	Cytokine			
Pawlowski et al.^16^	Gnotobiotic mouse	7–14 months, male			↑KC, IL-1β, & G-CSF (day 3) ↓IFN-γ, IL-12p40, IL-12p70, IL-10 (day 3)		
Van Opstal et al.^17^	Antibiotic cocktail	18 months (aged) vs. 8 weeks (young), male				Decreased serum anti-TcdA IgM (day 6), IgG (day 14), IgA (day 14)	↓ Bacteroidetes (day 3, 7, 14) ↓ Enterobacteriaceae (day 14) ↓ Firmicutes (day 0) ↓ Bacteroidetes:Firmicutes ratio (day 1, 3, 7)
Shin et al.^18^	Antibiotic cocktail	18 months (aged) vs. 8 weeks (young), male	↓ Neutrophil (tissue) (day 2)		↓ IL-1β, KC, MIP-2, TNF-α (day 2) ↑IL-1β (day 5)		↓ alpha-diversity (day 0) ↓ Bacteroidetes phylum (day 0)
Peniche et al.^19^	Antibiotic cocktail	12–14 month (aged) vs. 2–4 months (young), male and female	↓ Neutrophil (tissue) (day 7)		↓ IL-6, IL-23, IL-22 (day 7)		↑ alpha-diversity (day 0)
Abernathy-Close et al.^20^	Cefoperazone	22–28 months (aged) vs. 2–3 months (young), male and female	↓ Neutrophil (tissue) (day 2) ↓ Eosinophil (tissue) (day 2) ↓ Eosinophil (peripheral blood) (day 2)		↓ CXCL1 in serum (day 2) ↑ IL-17A in serum (day 2)		

## Gnotobiotic aged animal model of CDI

The first publication by Pawlowski et al. utilized an aged gnotobiotic mouse model of CDI.^16^ At the time, there was no established, widely accepted mouse model of CDI, and this was one of the first mouse models developed to study CDI.^16^ The golden Syrian hamster model had been in use traditionally for CDI research, but with 100% mortality and lack of diarrhea, the concern was the difference in presentation from human disease.^22,23^ In this model, gnotobiotic C57BL/6 mice aged 7–14 months were infected with UVA13, a clinical NAP1/BI strain of *C. difficile* isolated from a patient in University of Virginia Health Center, by oral gavage.^16^ Compared to mice orally gavaged with a nontoxigenic strain, all of the mice given toxigenic *C. difficile* developed diarrhea then died or became moribund requiring euthanasia within 72 hours.^16^ Infected mice showed significant tissue damage in both cecum and colon.^16^ The levels of proinflammatory cytokines KC, MCP-1, IL-1β, and G-CSF were higher than controls, while IFN-γ, IL-12p40, IL-12p70, and IL-10 were lower compared to controls.^16^

Interestingly, the neutrophilic infiltration, while present, was not remarkably elevated in the infected group, nor were the levels of some cytokines, including TNFα, IL-17, and IL-6, while the levels of IFN-γ, IL-12p40, and IL-12p70 were lower than uninfected controls.^16^ These findings hint at a more complex process than an excessive inflammatory response as the cause of the moribund state.

## Antibiotic-induced dysbiosis aged mouse models of CDI

### Antibiotic cocktail model

Due to the uniform mortality with CDI^16^ and to more closely reproduce CDI seen in humans, models utilizing specific pathogen-free mice challenged with antibiotics have been developed. A mouse model of utilizing a cocktail of four antibiotics in the water before an additional injection with clindamycin to produce susceptibility to infection, described in Chen et al.^24^ came to be one of the most widely utilized models of CDI.^25,26^ The model had several advantages: a model which presents the spectrum of findings in human infection, with mice showing diarrhea, weight loss, and death, but without 100% mortality, making it possible to study a range of severity of the disease. van Opstal et al. published the first paper applying this model in aged mice^17^ and compared aged (18-month-old) mice to young (8-week-old) mice head-to-head.^17^ The mice were infected following exposure to a four antibiotic (metronidazole, vancomycin, colistin, and gentamicin) cocktail in drinking water for 3 days followed by an intraperitoneal injection of clindamycin before orally infecting with 10^5^ CFUs of VPI10463, a laboratory strain of CDI that is highly toxigenic.^17^ This model showed a significantly worse outcome in aged mice compared to young mice in terms of weight loss and mortality but without 100% mortality.^17^ The model was modified for a relapse model, in which the mice were treated with vancomycin by oral gavage for 5 days after infection.^17^ More severe disease during recurrent infection in aged mice was observed, which also correlated with lower levels of antibodies compared to young mice.^17^ Aged mice produced lower levels of anti-toxin A IgG antibodies, while the use of antibiotics seemed to decrease antibody levels in both aged and young mice, suggesting a role of antibiotic in perpetuating the vicious cycle of recurrent CDIs.^17,27^

After establishment of the use of aged mice in a modified Chen mouse protocol, a follow-up study was performed which evaluated in more detail the innate immune response involved in CDI pathogenesis in the context of aging.^18^ In Shin et al., the difference in weight loss was observed again, with worse outcomes in aged mice.^18^ In this study, the innate immune response was analyzed in detail, looking at cellular and cytokine responses, which revealed a lower proinflammatory response early in the infection (day 2 postinfection) compared to young mice.^18^ There was lower neutrophil recruitment to the intestinal tissue, along with lower levels of IL-1β, TNF-α, KC, MIP-2, IFN-γ, and IL-17.^18^ In addition to analyzing the outcome and immune responses, the microbiome was analyzed in this study.^18^ The aged mice microbiota had low alpha-diversity, but also had lower numbers of bacteria in the Bacteroidetes phylum. Further microbiome analysis down to genus level, revealed three distinct genera: Alistipes, Bacteroides, and rc4-4,^18^ differentiating aged from young mice. These deficiencies in microbial composition were restored by FMT from young mice to aged mice.^18^ What is more impressive is that FMT led to an improvement in outcome and reversed the distinction between aged and young mice immune responses.^18^ These findings strongly suggest an important role of microbiome-mediated host response in CDI pathogenesis in aging.^18^

Another group of investigators (Peniche et al.) aged both male and female mice at their own institution,^19^ which allows evaluation of the interaction of sex and aging, and provides potentially a more controlled comparison for evaluation of the effect of aging on the microbiome. The infection protocol followed the Chen protocol,^24^ utilizing the antibiotic cocktail and infection with VPI 10463.^19^ The main difference from the previous models utilizing antibiotic cocktails was that middle-aged mice (12–14 month old) were used with young mice (2–4 months old) as controls.^19^ Again, higher weight loss, signs of infection, and mortality were observed in the older mice.^19^ Consistent with earlier studies, infection in the aged mice showed lower neutrophil infiltration into the intestinal tissue, along with lower IL-6, IL-23, CXCL2, CXCR2, and MPO.^19^ An important finding from this study was the notable role of IL-22 in outcome of infection.^19^ IL-22 production was decreased in aged mice as measured by both RNA expression and protein levels, and the effect of IL-22 was confirmed by protection and improvement in outcome conferred by administering recombinant IL-22.^19^ Microbiome analysis revealed a significantly lower Bacteroidetes and higher Proteobacteria, Enterococcaceae, and Pseudomonadaceae in aged mice.^19^ Interestingly, the alpha-diversity, meaning diversity in the composition of the microbiome, was higher in middle-aged mice in this study,^19^ which is the opposite of what was seen in Shin et al., which showed a lower diversity in aged mice.^18^ These findings/inconsistency is interesting because of the complexity of the microbiome. Since CDI is only possible in a disrupted microbiome,^21^ and normal microbiome has a suppressive effect on *C. difficil*e,^28^ a lower microbiome diversity is considered one of the factors predisposing to CDI, and has been associated with recurrences.^29^ However, it has been shown in human studies that the microbiome diversity generally tends to be higher in older individuals.^30^ As we will further elucidate in the discussions section, the specific members of the microbiome may be more important. This is also demonstrated in Shin et al., where the FMT from young mice led to a higher proportion of Bacteroidetes and protection, but no change in the diversity of the total microbiome.^18^

### Cefoperazone model

Mouse model of CDI utilizing cefoperazone alone to induce dysbiosis has also been developed.^31^ Abernathy-Close et al. utilized aged mice that were the oldest among the published studies (22–28 months old) and also bred in the same facility as young mice (2–3 months old).^20^ In addition to a different antibiotic treatment, two different strains, VPI10463 and 630, were used to explore the impact of aging on CDI outcomes by infecting with strains of different virulence.^20^ VPI10463, as described above, is a highly toxigenic strain that produces high levels of both toxin A and B, while 630, which is a clinical strain isolated from a patient, is considered low virulence due to the lower levels of toxin production.^20,31^ Both advanced age and strain VPI10463 were associated with more severe disease as demonstrated by clinical scores.^20^ Compared to infection with VPI10463, 630 resulted to mild symptoms and histopathologic scores were minimally changed and not different between young and aged mice.^20^ As was also seen in Shin et al.,^18^ the aged mice had lower histopathologic scores on day 2 postinfection when compared to young mice when infected with VPI10463.^20^ The neutrophil and eosinophil responses in the intestinal tissue in aged mice were significantly blunted compared to young mice, but there was actually a higher systemic eosinophil response with CDI in aged mice, which was not seen in young mice.^20^ In terms of the cytokine response, the aged mice had higher IL-17A but lower CXCL1 response compared to young mice.^20^ This study demonstrated consistent findings of the detrimental effect of aging on CDI in a different mouse model, with additional information on the eosinophil response, which has recently gained significant interest in binary-toxin producing strains.^32,33^

## Insight on CDI pathogenesis from the aged mouse models and application to human disease

### Neutrophil response

Innate immune response is considered the main immune response to CDI.^34^ High neutrophil count is associated with worse outcome to CDI,^35^ but neutrophil depletion has been shown to be associated with higher deaths from CDI in animal models.^36,37^ It is remarkable that across different aged mouse models utilizing different methodologies and different aged mice – Shin et al.:^18^ antibiotic cocktail model using NIA-aged mice; Peniche et al.:^19^ antibiotic cocktail model using investigator-aged mice; and Abernathy-Close et al.:^20^ cefoperazone model using investigator-aged mice – the neutrophil recruitment to infected tissue has been consistently seen to be depressed compared to young mice. Characterizing changes to the innate immune response with aging is complex. Studies to investigate the changes in neutrophil response with aging have not produced consistent results, with an increase in neutrophil recruitment to influenza infection in the lungs in a mouse model,^38^ no change of human neutrophils in migration *in vitro*,^39^ or a decrease of chemotaxis of human neutrophils *in vitro*,^40^ which makes the consistent trend with CDI even more striking. There are two questions that arise from these findings: (1) what caused the neutrophils to be lower in aged mice and (2) how do the lower neutrophils contribute to worse outcome in aged mice?

The difference in immune response to explain the lower neutrophil count in aged mice with CDI has not been elucidated yet. Chemokines such as KC (CXCL1) and MIP-2 (CXCL2) directly recruit neutrophils to infected tissue, while cytokines such as IL-1β, TNF-α, IL-17, IL-6, and IL-23 enhance inflammation.^41^ Shin et al. demonstrated lower levels of KC, MIP-2, IL-1β, and TNF-α on day 2 while Peniche et al. had lower levels of IL-6 and IL-23 on day 7.^18,19^ However, in Pawlowski et al., KC and IL-1β were higher on day 3.^16^ These contradictory findings suggest that recruiting chemokines or cytokines do not determine the age-related changes in neutrophil recruitment. Further research into the mechanism by which neutrophil recruitment is affected is needed.

The effect of the lower neutrophil recruitment is unknown at present. Human studies in CDI are more suggestive of elevated neutrophils as a risk factor for worse disease and higher deaths,^42,43^ so the beneficial effect of neutrophils in this context is somewhat unexpected. There are data in animal models showing that depletion of neutrophils prior to infection leads to higher deaths^44,45^ as well as some human studies showing neutropenia as a risk factor for worse outcome.^46,47^ These conflicting data suggest that the benefit or harm from neutrophils may be context dependent. We could hypothesize that neutrophils, of the right quantity recruited into the right location at right timing, is required for protection, and that the dysregulation of this response lead to adverse outcomes. Therefore the more specific question would be “what is the protective effect of neutrophils in the early stage of the infection?” Clearance of *C. difficile* by neutrophils is a consideration, but bacterial burden has been shown to be not associated with outcome in human disease.^48^ Indeed in Shin et al., the infection burden was not seen to be different in mice even when there is a significant difference in outcome from CDI.^18^ Other functions of neutrophil response such as immune regulation can be considered, and should be investigated further. As noted in tumor immunology, special neutrophils or new populations of cells such as, low-density neutrophils (LDN)^49^ and myeloid-derived suppressor cells (MDSC),^50^ may have immune-regulatory role but these cells have not been investigated in CDI.

### Eosinophil response

Eosinophils have been shown to be important in protection against CDI, especially when infected with *C. difficile* strains that produce binary toxin.^33,51^ Although the the protective effect of eosinophils was documented in these studies, the mechanism by which eosinophils protect the host from severe CDI has not been characterized. The protective effect of eosinophils has not been clearly demonstrated in the aged mouse as while there was lower number of eosinophils recruited to the intestinal tissue, the eosinophil count in the systemic circulation was actually higher in infected old mice in the studies by Abernathy-Close et al.^20^ The role of eosinophil recruitment to the intestinal tissue with CDI and effects of ILC2 and microbiota on this phenomenon in the context of aging is unclear.^20^

### IL-22 signaling

The IL-22 story is interesting in light of more recent studies pointing to a protective role of IL-22 in CDI pathogenesis.^52,53^ In Hasegawa et al., a change in the complement system decreased circulation of pathogenic Enterobacteriaceae during CDI,^52^ while in Nagao-Kitamoto et al., IL-22 was shown to modify intestinal microbiota to prevent growth of *C. difficile*.^54^ One of the key findings of Peniche et al. was the importance of IL-22 in the aged mice CDI outcome, demonstrating a lower production of IL-22 in aged mice, and protection of the aged mice when given recombinant IL-22 during infection.^19^ Since in Peniche et al., recombinant IL-22 was given on day 1 of infection, suggesting a host response-mediated effect on outcome rather than a microbiome-mediated effect. In an animal model of inflammatory bowel disease (IBD) it has been shown that IL-22 produced by neutrophils leads to beneficial barrier function by increased epithelial cell proliferation and mucus production and leads to recovery.^55^ IL-22 is part of the immune response associated with type 3 innate lymphoid cells (ILCs) during CDI.^56^ With the other finding from aged animal models of CDI being the importance neutrophil response, the IL-22 may point to an important and possibly protective role in the immune response associated with ILC3.

### Humoral response

Although innate immunity is thought to be the main immune response to an initial infection,^34^ antibody response to *C. difficile* toxins play an important role in recurrent infections.^5^ Bezlotoxumab, a monoclonal antibody against *C. difficile* toxin B (TcdB) has been shown to be effective in decreasing recurrent CDI in patients with CDI.^57^ Older age is one of the risk factors for more frequent recurrent CDI. In van Opstal et al. recurrent CDI was tested in an aged mouse model by treating infected mice with vancomycin for 5 days.^17^ Consistent with human disease, recurrence was observed few days after discontinuation of vancomycin, with fatal infection occurring more in the aged mice.^17^ This poor outcome correlated with the humoral response, as aged mice had lower serum anti-toxin A IgM levels on day 6 post infection, as well as lower IgG and IgA levels on day 14 post-infection.^17^ An additional finding is that vancomycin led to lower levels of antibody production without a difference in *C. difficile* infection burden, pointing to a potential role of the resident intestinal microbiome on regulating the antibody response.^58^ Recurrence leads to significant quality-of-life issues, especially in the older population, and thus, vaccination and potentially, microbiota-targeted strategies to enhance humoral responses in the elderly warrant further studies.

### Intestinal microbiota

The intestinal microbiota play an important role in CDI pathogenesis: disruption of the intestinal microbiome is a prerequisite for colonization by *C. difficile* because the normal microbiome has an inhibitory effect on *C. difficile* growth, referred to as “colonization resistance.”^21^ The fecal microbiome in aged individuals exhibited decreased colonization resistance compared to young subjects.^28^ Changes in microbiome with aging demonstrates a decreased relative abundance of Firmicutes phylum^59^ and a decrease in Bifidobacteria,^30^ but these studies showed a high individual variability. There are a number of beneficial organisms in *in vitro* and *in vivo* studies in protection against CDI such as *Clostridium scindens* which is suspected to play a role due to its unique bile acid metabolism,^60^ while bacteria in the *Lachnospiraceae* family have a suppressive effect on *C. difficile* as well.^61^ Changes in the composition of these organisms with aging have not been yet documented.

The microbiome was investigated in three of the studies, van Opstal et al., Peniche et al. and Shin et al.^17–19^ In Shin et al., in addition to a lower alpha-diversity in aged mice compared to young mice, there was a notable lower proportion of Bacteroidetes phylum in the aged mice, which were supplemented with FMT from young mice.^18^ The other major phylum, Firmicutes, was not different between young and aged mice.^18^ Random forest analysis revealed important genera, two of which (Bacteroides, Alistipes) belonged to Bacteroidetes.^18^ Interestingly, cage switching was the method for FMT in this study, which would lead to an exchange of the microbiome between the young and aged mice, but this did not lead to a significant change in the microbiome composition in young mice while the aged mice underwent significant changes and became closer to young mice.^18^ This would suggest that there was a deficiency in the microbiome of the aged mice that were supplemented by that from the young mice, and that the young mouse microbiome was more robust and resistant to change. Another interesting finding was that the microbiome difference between young and aged mice did not lead to a difference in *C. difficile* colonization, suggesting a mechanism other than colonization resistance in mediating the difference in outcome.^18^ Peniche et al. did not find any major differences between aged and young mice in terms of microbiome composition, although the alpha-diversity of the microbiota was significantly increased in the older mice compared with young controls,^19^ which is the opposite finding from Shin et al.^18^ Even though the two studies showed different results regarding the microbiome – Shin et al. showed the microbiome changing with aging while Peniche et al. did not show any differences in microbiome with aging – the difference in the innate immune responses, especially the neutrophil response, was consistent across different models.^18,19^ With only two studies looking at the microbiome, it is difficult to draw a conclusion on whether aging has a specific effect on the intestinal microbiome. However, we can conclude that the immune responses changes are seen with aging regardless of whether there is a microbiome difference with aging.

Does this mean that the changes with aging is not related to the microbiome? The answer is not so simple. In Shin et al., FMT from young mice lead to protection of aged mice from death by CDI and a change in its immune responses.^18^ CDI itself is a disease that only occurs in the presence of dysbiosis, and therefore it is plausible that there is a microbiome factor associated with aging. Further studies to evaluate aging effects on microbiome is needed.

Additional information from these studies regarding the microbiome is the dynamics of change of the microbiome with CDI in the context of aging. The main finding from Peniche et al. after infection was that OTU_11, the representative sequence of which demonstrated 99% identity to *C. difficile* by blast analysis against NCBI 16SrRNA gene database, was elevated in aged mice compared to young mice.^19^ Although this is not exactly analogous to the absolute *C. difficile* infection burden measured by qPCR of the *tcdB* gene as performed in Shin et al. and Abernathy-Close et al., it is interesting that the *C. difficile* infection burden measured in Shin et al. and Abernathy-Close et al. was not different between aged and young mouse groups,^18,20^ suggesting a different trend than Peniche et al.^19^ In van Opstal et al., the baseline microbiome in aged mice was not different from young mice in total numbers or numbers of Bacteroidetes and Enterobacteriaceae, while the Firmicutes were lower with aged mice.^17^ After infection with *C. difficile*, there is gradual decrease in overall bacterial numbers and the Bacteroidetes and Enterobacteriaceae, with Bacteroidetes significantly lower in aged mice especially lower with antibiotic treatment.^17^ There was an increase in Firmicutes numbers, likely reflecting the increase in *C. difficile* which is also a member of the Firmicutes phylum.^17^ The finding from this study that correlates with Shin et al. is the trend of the Bacteroidetes phylum^17,18^ Bacteroidetes numbers decreased with infection, but were even lower in aged mice, especially with vancomycin treatment.^17^ Bacteroidetes phylum is lower,^18^ or more susceptible to external stressors,^17^ which suggests the possibility of a protective function by members of the Bacteroidetes phylum against CDI, which is lost with age.

## Limitations of the current aged mouse models

An aged animal model poses significant challenges. Aged mice from the National Institute on Aging aged mice colony are limited in number, therefore the access has been limited to researchers with aging biology grants.^62^ Aged mice can be purchased from commercial laboratories, but is significantly higher in cost.^63^ Another option is to start an aged mouse colony, but this is also difficult due to the time and cost required. Aged mice utilized in the aged mouse models are around 12–24 months of age, necessitating maintaining the colonies for 1–2 years before any experiments can be performed, therefore considerable time and resources are committed before any results are seen. These difficulties raise the question of the utility of aged animal models of infection, whether the benefit seen from these studies outweigh the cost of the experiments.

The value of the aged animal model comes from the phenomenon that is being studied: aging. Even though aging is one of the most important factors to consider in CDI, it is also quite difficult to study due to the multifactorial nature of aging.^8^ There are many confounding factors that need to be considered, and cannot always be controlled in clinical studies.^8^ Utilizing an aged animal model allows us to control for some conditions, such as environmental factors and diet, to evaluate the effect of aging on pathogenesis of CDI. As aging is associated with worse outcomes in multitudes of illnesses including infections such as influenza^64^ and COVID-19,^65^ findings from utilizing the aged mouse model could have far-reaching applications in the study of aging. Another advantage of an aged mouse model of CDI is its use in testing therapeutic options against CDI, as the elderly will be the main target patient population to use new therapeutics in.

One finding from aged mouse model studies of CDI is the ability of the intestinal microbiome to lead to changes in disease outcome and immune responses in aged mice. Intestinal microbiome has always been considered important due to its effect on spore germination and colonization by the *C. difficile* bacteria.^28,60^ However, there have been studies linking the intestinal microbiome with immune response even in infections outside of the gastrointestinal tract.^66,67^ The finding that not only is the intestinal microbiome of the aged mouse distinct from that of the young mice, but also that transfer of the young mice microbiome into the aged mice reverses the effects of aging has major implications in our understanding of aging and immunity.^18^ This opens the doors to research on the interaction of the intestinal microbiome and immune response to a number of different conditions including various infections, malignancy, and autoimmune disorders. The possibility of microbiome-based therapeutics in improving health in the aging population would be relevant to commercial efforts for drug development.

## Research gaps and future directions

Current research gaps in the research of aging and CDI are quite large. The mechanism of action by which older age leads to worse outcome has not been elucidated. In the light of the COVID-19 pandemic, which affects older patients disproportionately,^68,69^ the physiologic and immunologic basis by which aging leads to worse outcome with infections becomes more important than ever as a research goal. At the present, aged animal models of CDI have been quite promising, showing consistent results of aging on CDI outcome as well as showing promising leads to investigate for mechanisms of aging: neutrophil or other inflammatory cell recruitment or activity, innate immunity, and antibody response. The interaction of microbiome with immune response is also demonstrated in Shin et al., which could be relatable to other infections and settings other than older age.^18^ That said, it is fair to say the research in this field is in its infancy, with significant gaps remaining in our understanding regarding the effect of aging on CDI. The effect of aging on the microbiome, the mechanism of action by which the microbiome modulates the immune response, the mechanism by which the relative neutrophil deficiency in the aged host leads to worse outcome, the role of IL-22 in the aging effect on CDI, the interaction of the microbiome and the humoral response, are some of the burning questions raised by these studies. In addition, studies to bring the knowledge back to older patients for translational research are needed.

## Data Availability

Data sharing is not applicable – no new data generated in this publication.
